# Antimicrobial stewardship and drug formulary restrictions during COVID-19: what is restricted and who decides?

**DOI:** 10.1017/ash.2023.205

**Published:** 2023-06-30

**Authors:** Alfredo J. Mena Lora, Rodrigo Burgos, Scott Borgetti, Lelia H. Chaisson, Susan C. Bleasdale

**Affiliations:** University of Illinois at Chicago, Chicago, IL, USA

**Keywords:** COVID-19, formulary restrictions, antimicrobial stewardship

## Abstract

COVID-19 therapies were challenging to deploy due to evolving literature and conflicting guidelines. Antimicrobial stewardship can help optimize drug use. We conducted a survey to understand the role of stewardship and formulary restrictions during the pandemic. Restrictions for COVID-19 therapies were common and approval by infectious disease physicians often required.

## Background

The COVID-19 pandemic presented a major challenge to healthcare systems worldwide. Throughout the pandemic, novel and repurposed drugs were used for the management of COVID-19.^1,2^ Significant disagreements emerged over optimal treatment for hospitalized patients with COVID-19 due to conflicting guidelines and rapidly evolving literature.^1,2^ Antimicrobial stewardship programs (ASPs) help optimize drug use by developing hospital guidelines, providing education, offering real-time feedback on prescribing practices, and implementing drug formulary restrictions.^3^ Thus, ASPs are uniquely positioned to support pandemic preparedness and response when novel treatments emerge.^3,4^

Drug formulary restrictions are an important stewardship strategy that have been used to ensure that antimicrobials and COVID-19 therapies are used in accordance with the latest evidence and guidelines.^3,5^ However, there is a paucity of data on the role of ASP and formulary restrictions for COVID-19 therapeutics. In this study, we sought to understand and describe the role of stewardship and drug formulary restrictions in US hospitals during the COVID-19 pandemic.

## Methods

### Study design and participants

We conducted a cross-sectional survey to assess the role of drug formulary restrictions at US hospitals. A web-based survey was developed in REDCap to capture data on characteristics of survey respondents, hospitals represented, and formulary restrictions for commonly used therapies for hospitalized patients with COVID-19 requiring supplemental oxygen and noninvasive or invasive ventilation (Supplement 2). Remdesivir, convalescent plasma, and IL-6 and JAK-2 inhibitors were included in our survey. Hydroxychloroquine and ivermectin were excluded due to lack of emergency use authorization by the FDA. Treatments for nonhospitalized patients, such as monoclonal antibodies and oral antivirals, were excluded. On April 22 and 25, 2022, we recruited participants by distributing a survey via online messaging boards and listservs for members of the Infectious Diseases Society of America (IDSA) IDea network, IDSA Antimicrobial Stewardship Centers for Excellence, and the Society for Healthcare Epidemiology. The survey evaluated restrictions throughout the pandemic and remained open through May 15, 2022. Participants submitted responses anonymously.

### Analysis

We assessed characteristics of survey respondents and respondents’ hospitals, the proportion of hospitals with formulary restrictions in place at any time during the COVID-19 pandemic, and specialties of providers authorized to place orders. In addition, we compared characteristics of drug formulary restrictions in hospitals where infectious diseases (IDs) physicians were authorized to place orders versus those where non-ID providers were authorized.

### Ethics approval

The University of Illinois at Chicago Institutional Review Board approved this study.

## Results

### Survey respondents

A total of 99 survey responses were received, of which 4 hospitals outside the US were excluded. Of the 95 surveys included, 28 states and 44 cities were represented (Supplement 1). Survey respondents included 51 (54%) ID physicians, 37 (39%) pharmacists, 2 (2%) hospitalists, and 5 (5%) infection preventionists. Characteristics of hospitals with and without stewardship are listed on Table 1.

**Table 1.** Hospital characteristics

	All N = 95	With ID approval N = 77	Without ID approval N = 18
Hospital type			
Community	35 (37%)	26 (34%)	5 (28%)
County	3 (3%)	3 (4%)	0 (0%)
University	47 (49%)	32 (42%)	10 (56%)
University-affiliated	10 (11%)	10 (13%)	1 (5%)
Other	0 (%)	6 (8%)	2 (11%)
Hospital size			
<200 beds	14 (15%)	8 (10%)	6 (33%)
201–300 beds	15 (16%)	13 (17%)	2 (11%)
301–400 beds	14 (15%)	14 (18%)	0 (0%)
>400 beds	52 (54%)	42 (55%)	10 (56%)

### Stewardship

**Figure 1.**
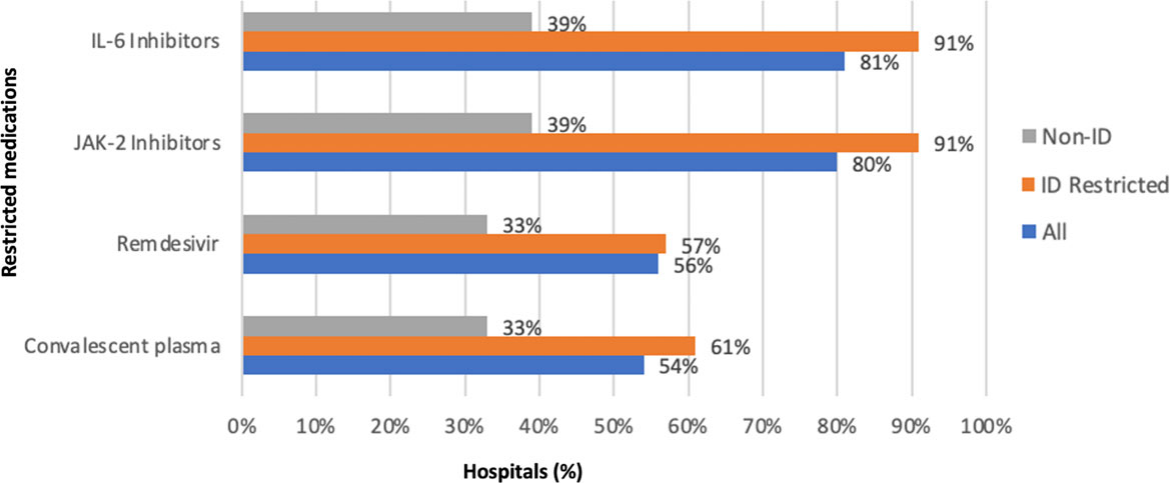
Restricted COVID-19 therapies by medication type (%) in all facilities and facilities with or without ID physicians ordering or approving restricted medications.

All hospitals but one hospital had ASP (n = 94, 99%) and 91 (96%) had formulary restrictions for COVID-19 therapies. Hospitals reported formulary restrictions for IL-6 inhibitors (n = 77, 81%), JAK-2 inhibitors (n = 76, 80%), remdesivir (n = 53, 56%), and convalescent plasma (n = 41, 54%) (Fig. 1). Restricted medications could be ordered or approved by ID physicians (n = 77, 81%), pulmonary/critical care physicians (n = 43, 44%), pharmacists (n = 27, 28%), designated hospitalists (n = 12, 13%), and designated COVID-19 team of providers of any discipline (n = 16, 17%) in hospitals with formulary restrictions. Hospitals where restricted medications could only be ordered or approved by ID physicians had more drug formulary restrictions (Fig. 1). Hospitals where ID physicians were not involved in the ordering or approval of restricted medications (n = 18, 19%) reported less formulary restrictions for IL-6 inhibitors (n = 7, 39%), JAK-2 inhibitors (n = 6, 33%), remdesivir (n = 6, 33%), and convalescent plasma (n = 7, 39%) (Fig. 1).

## Conclusion

In our survey, formulary restrictions for COVID-19 therapeutics were commonly reported (n = 94, 96%) and approval by ID clinicians was commonly necessary (n = 77, 81%). The role of other specialties was significantly lower, with pulmonary/critical care as a distant second (n = 43, 44%). Though restrictions were present for most therapies, they were more commonly used for immunomodulators. Hospitals where ID physicians did not order or approve restricted medications had more unrestricted use of these medications. In smaller hospitals, this may reflect lack of access to ID physicians. Formulary restrictions may be a crucial tool for pandemic response in that they ensure access to medications to those who need them when availability is limited. A wider gap between remdesivir use and guideline concordance in hospitals without formulary restrictions has been reported.^6^ Our survey highlights the value of ID clinicians to healthcare systems in allocating resources and promoting evidence-based practices during the COVID-19 pandemic.

Several obstacles made it challenging to bring novel COVID-19 therapies to the bedside. Rapidly evolving literature and conflicting guidelines often led to differences in the management of COVID-19.^1,2^ ASP can help standardize therapy, inform clinicians of changing guidelines and novel therapies, and align treatments with evidence-based guidelines.^3,4^ Supply constraints and cost also make stewardship imperative. During the initial months of the pandemic, there was high demand for remdesivir and limited supply.^7^ Similarly, tocilizumab was in short supply at various points of the pandemic due to very high demand.^8^ High cost and demand at a time when healthcare systems are under financial pressure by other aspects of the pandemic, such as decreased revenue and rising staffing costs, make stewardship a crucial part of pandemic response.^9^ Remdesivir, for example, costs approximately $520 per vial, totaling over $2,000 per course.^10^ Similarly, tocilizumab and baricitinib cost approximately $3,625 and $2,326, respectively, when used for COVID-19.^10^ Thus, stewardship is vital to ensure the evidence-based use of pharmaceuticals that may be both expensive and in limited supply. The high cost and supply constraints of immunomodulators may be contributing factors to why these agents were more commonly restricted.

ID physicians have been at the frontlines of pandemic preparedness and response, often taking on multiple roles. These roles include providing clinical care, conducting clinical research, providing advice on infection prevention and control, influencing public policy, educating the public, and creating treatment protocols and management plans for COVID-19 patients. Our survey highlights the contribution of ID physicians in the stewardship of expensive and at times limited supply of medications. At a time with rampant disinformation and significant therapeutic controversies, ASP and ID physicians have been instrumental in ensuring that these medications are used appropriately, based on clinical guidelines and in accordance with the principles of stewardship. Indeed, the pandemic has further highlighted the value of ID physicians and of collaborative multidisciplinary stewardship teams.

Our study has several limitations. Our convenience sample was small, and ID physicians and pharmacists may have been more likely to participate due to our use of ID society listservs. Our survey did not capture specific hospital guidelines or timing of the formulary restrictions. Nonetheless, our study offers important knowledge on stewardship strategies and ID physician roles ring the COVID-19 pandemic.

Emerging and re-emerging infections will continue to pose challenges for clinicians. Lessons learned during the COVID-19 pandemic may help us prepare for future challenges. In this survey, we found that ASP and drug formulary restrictions were a common strategy during the COVID-19 pandemic. The important role played by ASP and ID physicians in the stewardship of expensive medications during the COVID-19 pandemic highlights the value of ID in yet another critical area of pandemic response.

## Supplementary material

For supplementary material accompanying this paper visit http://doi.org/10.1017/ash.2023.205. click here to view supplementary material
