# Supplementary figures and images for: Antimicrobial stewardship and drug formulary restrictions during COVID-19: what is restricted and who decides?
Source: Antimicrob Steward Healthc Epidemiol. 2023 Jun 30;3(1):e116. doi: 10.1017/ash.2023.205 (PMC10369429; doi:10.1017/ash.2023.205)

Supplement 1. Geographic distribution of hospitals represented in the survey


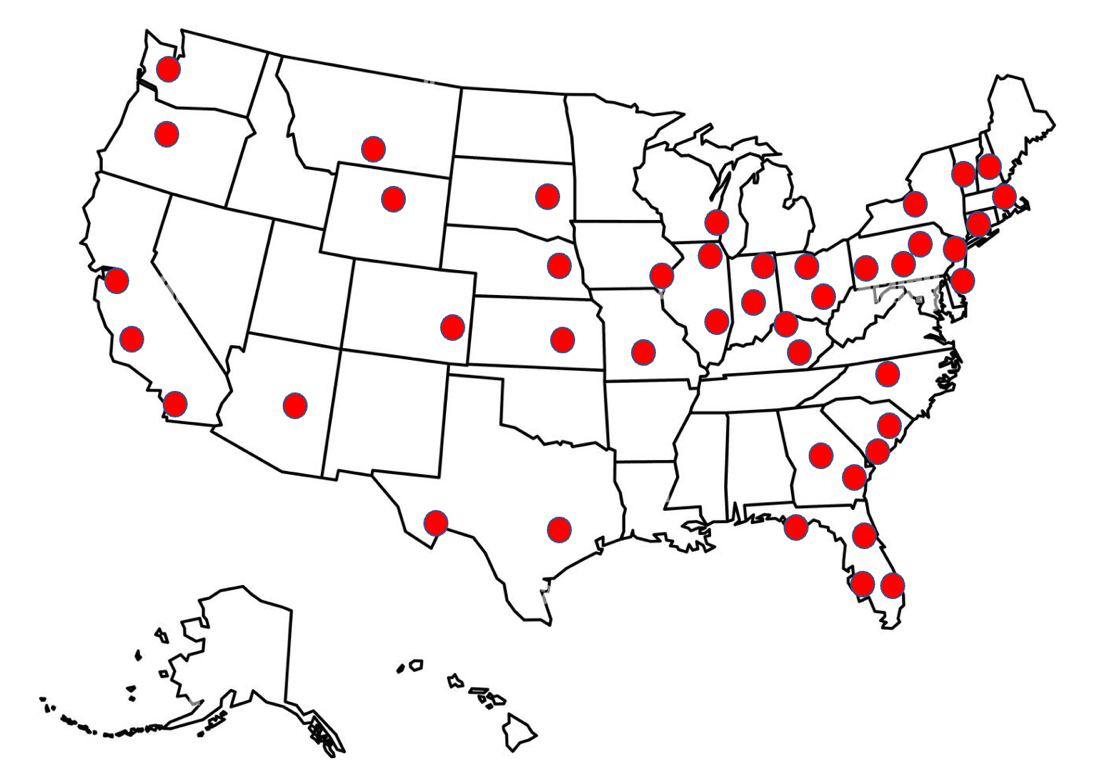

Supplement: Supplementary file 1 [file ashsup.zip › S2732494X2300205Xsup001.docx]

Supplement 2. Survey Tool


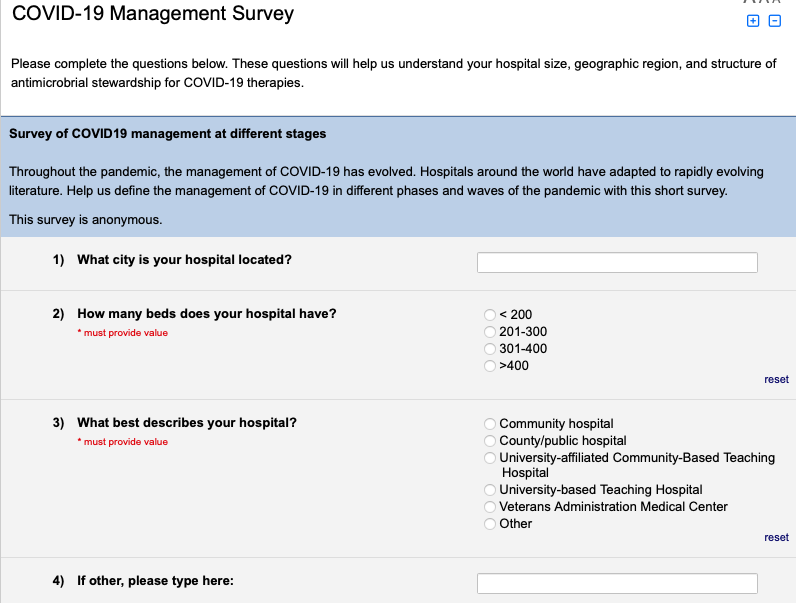

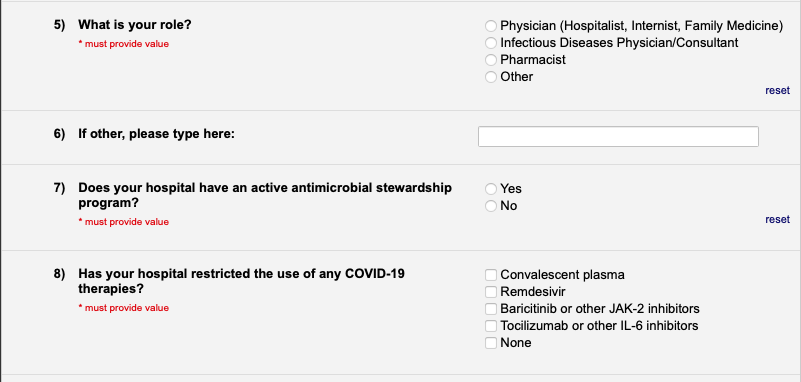

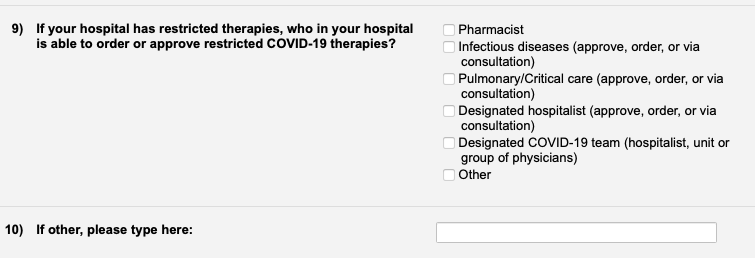

Supplement: Supplementary file 1 [file ashsup.zip › S2732494X2300205Xsup002.docx]
